# Method of determining technique from weight and height to achieve targeted detector exposures in portable chest and abdominal digital radiography

**DOI:** 10.1002/acm2.13582

**Published:** 2022-03-09

**Authors:** Matthew Hoerner, Kevin Grizzard, Jennifer Moroz

**Affiliations:** ^1^ Department of Radiology and Biomedical Imaging Yale University and Yale New Haven Hospital New Haven Connecticut USA; ^2^ Department of Radiology and Biomedical Imaging Yale New Haven Hospital New Haven Connecticut USA; ^3^ Department of Radiology Brigham and Women's Hospital Boston Massachusetts USA

**Keywords:** EI, portable, technique, X‐ray, chart

## Abstract

This study presents a methodology to develop an X‐ray technique chart for portable chest and abdomen imaging which utilizes patient data available in the modality worklist (MWL) to reliably achieve a predetermined exposure index (EI) at the detector for any patient size. The method assumes a correlation between the patients’ tissue equivalent thickness and the square root of the ratio of the patient's weight to height. To assess variability in detector exposures, the EI statistics for 75 chest examinations and 99 abdominal portable X‐ray images acquired with the new technique chart were compared to those from a single portable unit (chest: 3877 images; abdomen: 200 images) using a conventional technique chart with three patient sizes, and to a stationary radiography room utilizing automatic exposure control (AEC) (chest: 360 images; abdomen: 112 images). The results showed that when using the new technique chart on a group of portable units, the variability in EI was significantly reduced (*p* < 0.01) for both AP chest and AP abdomen images compared to the single portable using a standard technique chart with three patient sizes. The variability in EI for the images acquired with the new chart was comparable to the stationary X‐ray room with an AEC system (*p* > 0.05). This method could be used to streamline the entire imaging chain by automatically selecting an X‐ray technique based on patient demographic information contained in the MWL to provide higher quality examinations to clinicians by eliminating outliers. In addition, patient height and weight can be used to estimate the patients’ tissue equivalent thickness.

## 1 INTRODUCTION

X‐ray technique selection in portable imaging can be a challenging hurdle in the ongoing effort to maintain image quality across a wide range of patient sizes.^1^, ^2^, ^3^, ^4^, ^5^ Technologists have mostly been educated to operate digital detectors by targeting a single detector exposure level to reduce noise and avoid saturation.^6^, ^7^, ^8^, ^9^ Digital detector image quality is primarily determined by quantum mottle, while global image contrast is maintained over a wide range of detector exposures. This can create complacency and result in a wide range of exposures that still produce readable examinations.^8^ In some cases, images may be under‐exposed because the patient was considerably larger than the typical patient for which a programmable technique is designed. Yet producing detailed technique charts that may require measuring the patient's thickness can result in charts that are cumbersome to read and implement, and which therefore lead to low compliance from technologists. Radiographic systems that utilize automatic exposure control (AEC) should have less variability since they are able to monitor exposure after the X‐ray beam has traversed the patient, but of course these are not available on portable systems. Furthermore, AEC systems in radiography are typically unable to distinguish between primary and scatter radiation, which can result in reduced image contrast if the kVp or mAs is not increased for larger or denser patients. Therefore, a system that can compensate for contrast reduction due to scatter would be ideal for thicker patients.^10^, ^11^, ^12^

Given the advent of sophisticated Radiology Information Systems (RIS), more information can be communicated to the technologist acquiring a portable X‐ray image than in the past. For example, patient height, weight, age, and study indication are all present on requisition forms. This information can in turn be leveraged to determine techniques more tailored to individual patients without placing an excessive burden on technologists.

The goal of this study is to develop an approach to utilize RIS information in selecting an appropriate X‐ray technique for portable examinations of the abdomen and chest by modeling the tissue equivalent thickness of the patient based on information contained in the study order. This approach aims to reduce the overall variability of detector exposures in portable imaging by considering patient height and weight, the typical imaging geometry for a given examination type, whether an anti‐scatter grid is used, and tube output characteristics.

## 2 METHODS

Studies have shown that technique charts can utilize a doubling thickness, defined as the thickness of tissue that requires doubling the mAs, to maintain exposure at the detector. The doubling thickness is often between 2.5 and 3.5 cm body thickness.^2^, ^13^, ^14^ The use of a doubling thickness would indicate that the X‐ray beam can be modeled using the narrow‐beam attenuation model. Estimation of air kerma to the detector for a diagnostic examination requires knowledge of the air kerma incident on the detector without attenuation through the patient, the patient's tissue equivalent thickness, the beam quality, and the attenuation characteristics of the patient for the given quality X‐ray beam. The exposure index (EI) can be used as a surrogate for the incident air kerma at the detector, although this relationship is dependent on beam quality. The EI is defined as a unit‐less quantity by the International Electrotechnical Commission (IEC) to be equal to the incident air kerma (in micro‐Gray) to the detector multiplied by 100 from a spectrum that represents RQA‐5 beam quality.^15^ The incident air kerma at the detector without attenuation, for a given tube current‐time product (mAs), is determined by placing a dose measuring device at a reference distance from the tube and acquiring a series of measurements at different tube potentials (kVp). This provides the air kerma per mAs (tube output) as a function of kVp. Knowing the response of tube output with kVp and using the inverse‐square law, one can approximate the air kerma at any point in space along the central axis, for any kVp, source‐to‐image distance (SID), and mAs combination. Beam quality, which is often characterized by the half‐value layer (HVL) of aluminum, can be easily determined either with a solid‐state dose probe or by using aluminum sheets iteratively.

The incident air kerma at the detector depends on the effective attenuation coefficients for the given beam quality and on the tissue equivalent thickness of the patient, which progressively hardens and attenuates the beam with increasing thickness. The effective attenuation coefficients were calculated for various X‐ray spectra from portable radiography X‐ray tubes for ICRU‐44 soft tissue using a spectrum generator code, SPEKTR.^16^, ^17^ SPEKTR generates a tungsten anode spectrum based on specification of the first and second HVL. A soft tungsten anode spectrum is hardened in an iterative fashion until the theoretical HVL matches the measured one. For our spectrum, we used the first HVL as a matching value. The resultant spectrum is assumed to approximate the X‐ray spectrum incident on the patient. The SPEKTR program has been used in a wide range of applications for X‐ray source modeling.^18^, ^19^ The calculated X‐ray tube spectra were then filtered by ICRU‐44 soft tissue in 1 keV energy bins for a range of thicknesses to compute the effective attenuation coefficient.^20^

The X‐ray spectra were generated for various beam qualities specific to our portable X‐ray machines and used to evaluate the effective linear‐attenuation coefficients from the NIST standard reference database in soft tissue (ICRU‐44) for depths of 0 through 60 cm in 15 cm increments. These effective linear‐attenuation coefficients were compiled in a table based on X‐ray tube beam quality expressed in HVL of aluminum and soft tissue thickness to be used for evaluating X‐ray beam attenuation in patients.

The patients’ water‐equivalent diameter (WED) was estimated assuming that the patient can be represented by a water‐filled cylinder of height *H* (mm) and volume *V* (mm^3^), where

(1) $$V\;=\;\pi {\left(\frac{\mathrm{WED}}{2}\right)}^{2}\;H$$

Since weight (kg) is given by the product of water density and volume,

(2) $$W\;=\rho_{\mathrm{water}}\;\;V,$$

the diameter of such a cylinder can be calculated using

(3) $$\mathrm{WED}\;=\sqrt{\frac{4}{\pi \rho_{\mathrm{water}}}\frac{W}{H}}\;\;\underline{\underline{\mathrm{def}}}\;\;C\sqrt{\frac{W}{H}}$$

*C* is a constant equal to about 1130 $mm^{\frac{3}{2}}kg^{-\frac{1}{2}}$ for water (density of 10^–6^ kg/mm^3^). This expression for WED is very similar to the relationship defined by Ogawa.^21^

Validation of this equation and determination of the value of *C* was performed by fitting the patient's WED to values of (*W*/*H*)^1/2^ calculated from patient's demographic data available in the RIS. The WED was measured on computed tomography (CT) images by a CT dose monitoring application Radimetrics™ (version 3.0, Bayer HealthCare LLC, Whippany, NJ) in accordance with AAPM Report 220.^22^ Since patients are not actually cylindrical, it is expected that the coefficients in such a fit might vary based on body region, so this process was done independently for chest (*n* = 235) and abdomen (*n* = 263) examinations.

Attenuation through a patient, of diameter WED, may be expressed by

(4) $$\mathrm{AF}\approx \exp\left(-\bar{\mu_{w,Q}}*\mathrm{WED}\right),$$

where AF is the attenuation factor and $\bar{\mu_{w,Q}}$ is the effective attenuation coefficient of water for the beam quality *Q* hardened by WED. However, a more direct relation to the patient attenuation can be established. The actual attenuation factor from the patient is

(5) $$\mathrm{AF}\;=\;\exp\left(-\bar{\mu_{t,Q}}*t\right),$$

where *t* is the tissue equivalent thickness and is dependent on both the body part and projection orientation. $\bar{\mu_{t,Q}}$ is the effective linear‐attenuation coefficient for the beam quality *Q* for this tissue equivalent thickness. Recall that since the patient progressively hardens the beam, $\bar{\mu_{t,Q}}$ has a dependence on tissue thickness.

The X‐ray tube output measurements were used to evaluate the tissue equivalent thickness (*t*) using the following equation:

(6) $$t\;=\frac{\ln\left(\frac{\mathrm{output}\left(kVp\right)\times \mathrm{mAs}\times \frac{{d_{ref}}^{2}}{\mathrm{SI}D^{2}}}{\frac{\mathrm{EI}}{c_{0}}}\right)}{\bar{\mu_{t,Q}}\left(\mathrm{HVL},\mathrm{WED}\right)}$$

In our measurements, *d*_ref_ is 70 cm, which is the source‐to‐detector distance used during our annual physics testing. The SID used for the portable images was 100 cm (abdomen) and 127 cm (chest). The factor *c*_o_ is used to convert EI to air kerma and is defined as 100 μGy^–1^.^15^ Output as a function of kVp and HVL data was collected from every portable unit using a solid‐state probe (Raysafe X2 R/F Probe, Unfors RaySafe AB, Billdal, Sweden). The IEC EI under RQA‐5 conditions was also verified on each detector for accuracy to within 10%.

The relationship between tissue equivalent thickness defined in Equation (6) and WED defined in Equation (3) was determined experimentally by collecting data consisting of (*n* = 171) portable non‐grid chest and (*n* = 63) portable with anti‐scatter grid abdomen images. A linear fit produced a relationship *t*(WED), giving tissue equivalent thickness as a function of WED. The latter is determined by a fit to weight and height. Images originated from a variety of models of portable X‐ray units from different facilities, along with image technical parameters accessed through the image DICOM header: kVp, mAs, EI, patient height (*H*), and patient weight (*W)*. Portable anteroposterior (AP) chest images acquired on five portable X‐ray units (Carestream DRX‐Revolution, Carestream Health, Rochester, NY) over a 1‐month period were used to obtain a fit of the estimated AP chest tissue equivalent thickness as a function of patient WED. Portable AP abdomen images acquired on five portable X‐ray units (Shimadzu MobileDaRt, Shimadzu Corporation, Kyoto, Japan) over a 3‐month period were used to evaluate the estimated AP abdomen tissue equivalent thickness versus patient WED. Based on the relationship of tissue equivalent thickness and WED, one can solve for the mAs required for a patient's H and W, given a kVp, HVL, target EI (EIt), and X‐ray tube output (μGy/mAs):

(7) $$\mathrm{mAs}=\frac{\left(\frac{EI_{t}}{c_{0}}\right)\times e^{\bar{\mu_{t,Q}}\times t\left(\mathrm{WED}\right)}}{\mathrm{output}}\;\times \frac{\mathrm{SI}D^{2}}{d_{\mathrm{ref}}^{2}}$$

For a linear function of output versus kVp with fitting parameters *c* and *d*, the required mAs is defined as:

(8) $$\mathrm{mAs}=\frac{\left(\frac{EI_{t}}{c_{0}}\right)\times e^{\bar{\mu_{t,Q}}\times t\left(WED\right)}}{c\times kVp+d}\times \frac{\mathrm{SI}D^{2}}{d_{\mathrm{ref}}^{2}},$$

where the equation for output in μGy per mAs is used to solve for mAs and corrected for distance. The proposed technique charts used a kVp range between 75 and 85 for portable AP abdomen and 70 and 95 for portable AP chest. The range in kVp of the new technique chart matched the range of kVp of the old technique chart and the data used to derive the tissue equivalent thickness. At our facility, portable chest exposures are performed without a grid, while portable abdomen images are acquired with an anti‐scatter grid (5:1 ratio, 103 lines per inch).

EI values from the old technique chart were extracted using tools created by both vendors that allow users to export data from each image taken. This resulted in 3877 portable AP non‐grid chest images from a single portable unit (Rev2) using a conventional technique chart, 360 PA chest images acquired on an upright Bucky system with AEC (Fixed Room A), 200 portable AP abdomen images acquired with a grid from a single unit (Dart5) using a conventional technique chart, and 112 abdomen images acquired using a table Bucky with AEC (Fixed Room A).

The new technique charts were created for abdominal (with anti‐scatter grid) and chest (non‐grid) portable AP X‐rays. For a selected kVp and patient height/weight combination, the mAs necessary to achieve the target EI was determined using Equation (8). The target EI for portables of the torso at our institution is 300. The kVp was modified based on tissue equivalent thickness to prevent long exposure times.

**FIGURE 1:**
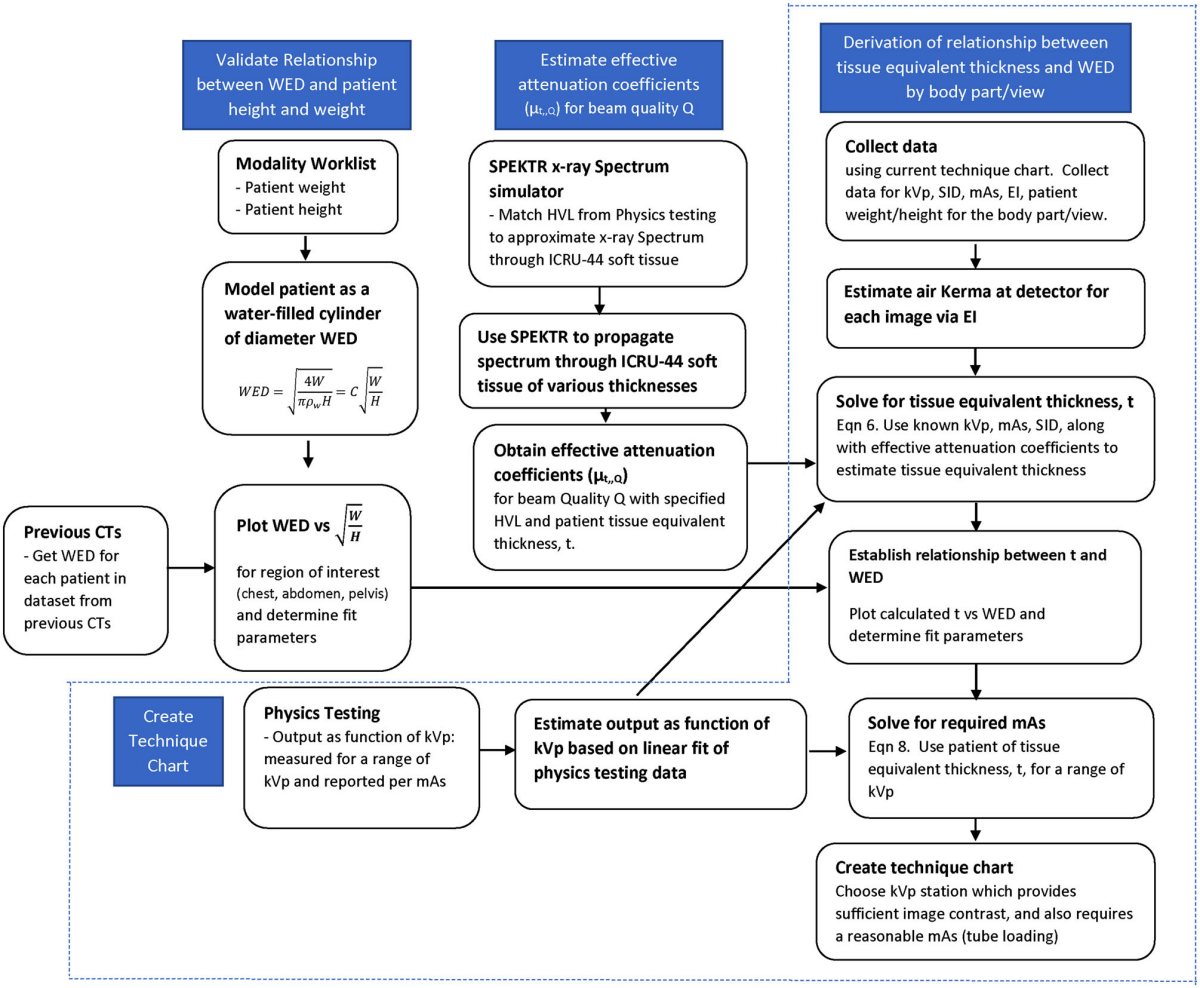
Flowchart describing the methodology of technique chart construction. The left column describes the formulation of the relationship between patient height and weight into water‐equivalent diameter (WED). The center column describes how effective attenuation coefficients are computed from half‐value layer (HVL) measurements and the SPEKTR program.^17^ The bottom row outlines the physics measurements required for each portable unit or the tube output characteristics needed to summarize the output as a function of kVp for a model portable unit. These relationships are used to compute tissue equivalent thickness (*t*) in the third column. The relationship of height and weight and the tissue equivalent thickness can be solved for each anatomical projection. This relationship along with the tube output characteristics are used to compute the required mAs for a desired kVp and target exposure index (EI) in the technique chart as a function of patient height and weight. The dotted blue lines contain the steps required to use this methodology for future creation of technique charts

A total of 75 chest studies were performed using the new chest technique chart and 99 abdomen studies were performed using the new abdomen technique chart. Variation in EI was determined and compared to portable chest and abdominal examinations performed on a single portable unit using the old technique chart with three patient sizes (small, medium, and large), from which the technologist selects the technique based on the patients’ physical appearance. A second comparison was made against a single stationary X‐ray unit using a Bucky system that incorporates an AEC system. Levene's absolute test was performed to access the variation in EI. All linear fits were determined using Pearson correlation and by applying the *t*‐test for significance. Figure 1 summarizes the methods and measurements required to build each examination technique chart. The final technique chart is reconstructed in tabular format. The rows represent patient weight and columns represent patient height. The data in the table contain the derived kVp and mAs.

## 3 RESULTS

The effective linear‐attenuation coefficients are presented in Table 1. The effective linear‐attenuation coefficient was interpolated from the data in Table 1 using an initial estimate of tissue equivalent thickness based on patient WED and beam quality. For our technique charts and typical patient thicknesses, this would be equivalent to a doubling thickness between 2.8 and 3 cm.

**TABLE 1:** Effective linear attenuation coefficients (cm^–1^) for ICRU‐44 soft tissue as a function of half‐value layer (HVL)

HVL (mm Al)	Thickness (15 cm)	Thickness (30 cm)	Thickness (45 cm)
2	0.307	0.288	0.278
2.44	0.276	0.260	0.251
2.81	0.257	0.243	0.235
3.2	0.246	0.233	0.225
3.63	0.233	0.222	0.216
4.03	0.226	0.216	0.210
4.42	0.221	0.211	0.205
4.76	0.216	0.207	0.201

**FIGURE 2:**
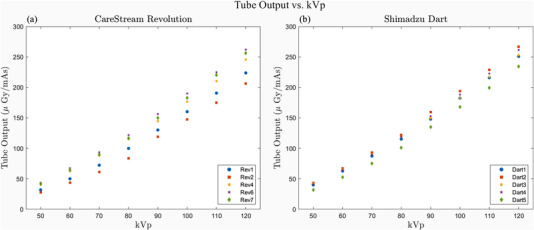
Tube output along the central axis of the X‐ray beam expressed in air kerma per tube‐current product as a function of tube potential for five Carestream Revolution portables used to acquire chest X‐rays (A). Tube output for five Shimadzu MobileDaRt portables used to acquire abdomen X‐rays is shown in (B). Measurements were acquired 70 cm from the focal spot

The relationship between tube output as a function of tube potential (kVp) is shown in Figure 2. This relationship is dependent on the total filtration of the X‐ray system and hardening from the anode angle, so it must be evaluated on a unit‐by‐unit basis. The results show some variability in output for units of the same make and model. The relationship was adequately modeled with a linear function over the kVp range investigated (*p* < 0.01).

**FIGURE 3:**
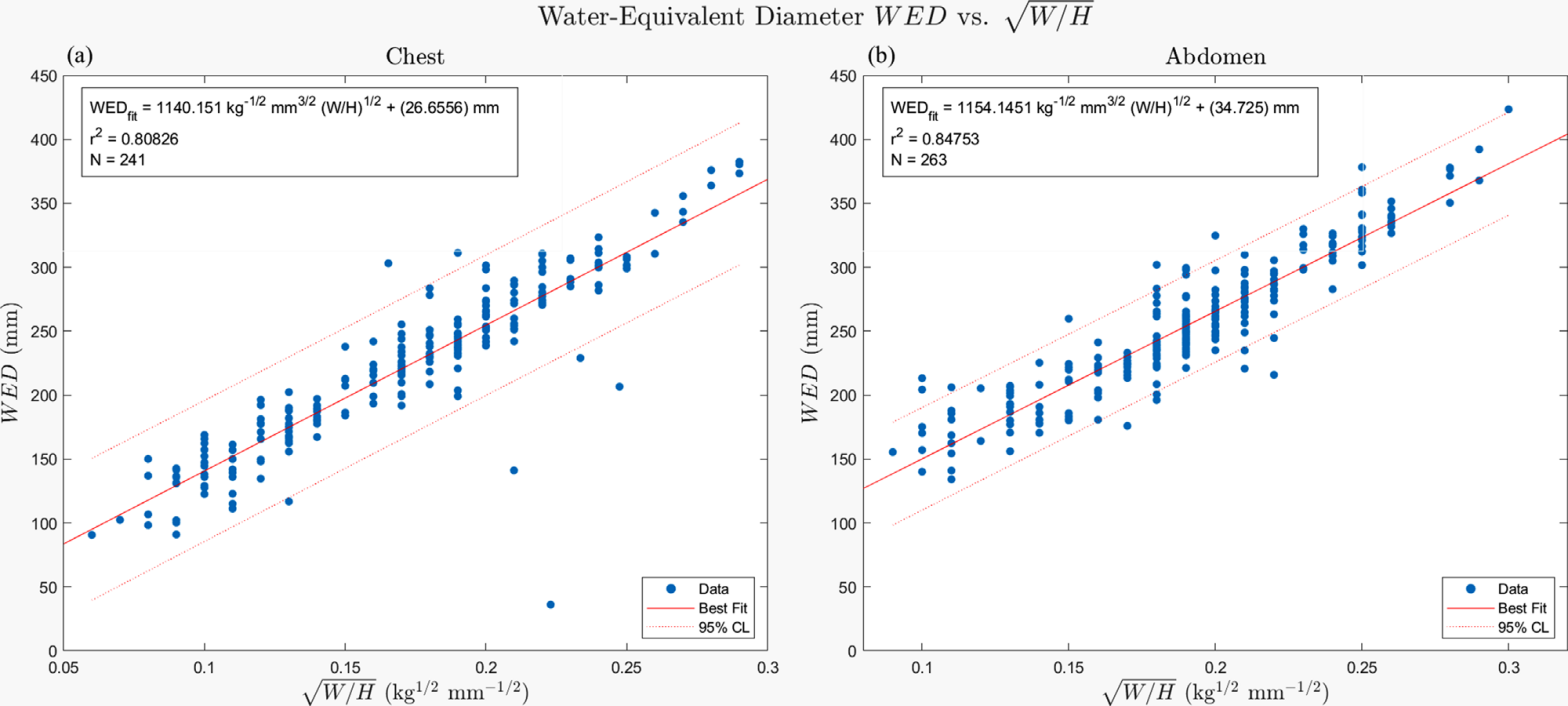
Body region water‐equivalent diameter (WED) as a function of patient height (H) and weight (W) for chest computed tomography (CT) examinations (A). Relationship for abdominal CTs is shown in (B). Note that the slope for each case is close to the theoretical value of 1130 mm^3/2^ kg^–1/2^ for water

The relationship between patient height and weight and WED for all chest (*n* = 241) and for all abdomen/pelvis (*n* = 245) CT studies is shown in Figure 3A,B. The slope for abdominal CT examinations (1154) and slope for chest examinations (1140) are both comparable to the theoretical value (1130 $mm^{\frac{3}{2}}kg^{-\frac{1}{2}})$. The relationship is linear, as our model predicted, with a Pearson correlation coefficient of 0.964 (*p* < 0.01) and 0.925 (*p* < 0.01), respectively. The dependence of WED on height and weight indicates that the relationship is similar for these anatomic regions, which simplifies our assessment of WED in the torso region.

**FIGURE 4:**
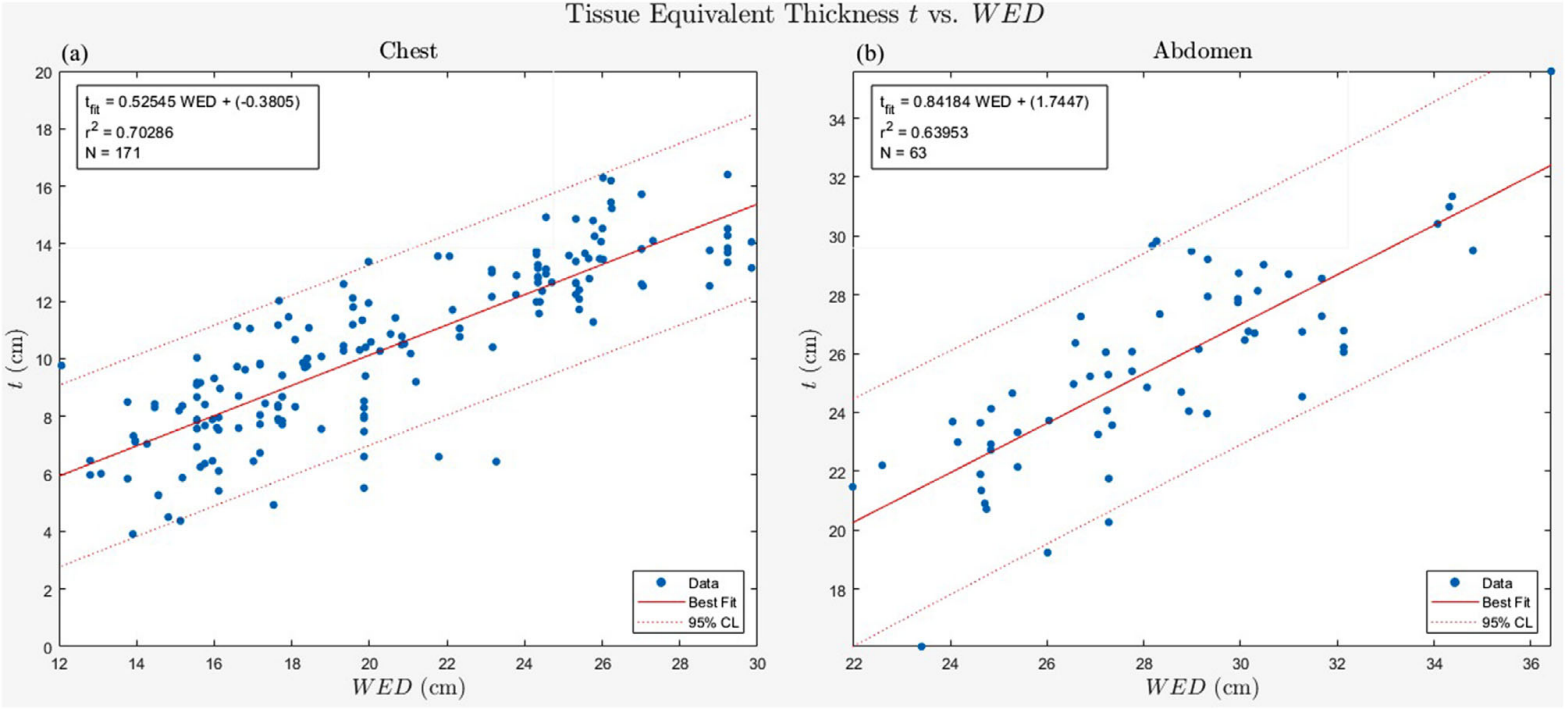
Body part tissue equivalent thickness (*t*) as a function of water‐equivalent diameter (WED) for chest X‐rays (A) and abdomen X‐rays (B). Dotted line represents the 95% confidence level in the fit. The tissue equivalent thickness was determined using Equation (6). WED diameter was calculated for each patient based on their height and weight along with the relationship in Figure 3

Figure 4 illustrates the relationship between tissue equivalent thickness and the WED. These relationships demonstrate the difference of chest versus abdominal portable examinations and how tissue equivalent thickness depends on the patient, body part, and projection. The results indicate that the AP chest tissue equivalent thickness is about 53% of WED, while the AP abdominal tissue equivalent thickness is 85%. The AP abdominal tissue equivalent thickness is consistent with observations from the AAPM Task Group 220 report.^22^ The AAPM data from Table 1B and 1C indicates a ratio of 0.85 when solving for the ratio of AP dimension length to patient WED across all diameters presented in the table. A study by Burton et al. showed the ratio of AP to effective diameter in the abdomen to be 0.85 and 0.79 in the chest.^23^ A significant positive intercept in these fits indicate additional attenuation external of the patient. The abdominal portables were acquired using an anti‐scatter grid that attenuated both primary and secondary photons, which would explain the positive intercept. A positive intercept of 1.7 cm would result in a Bucky factor of approximately 1.5. The relationship is linear with a Pearson correlation coefficient of 0.838 (*p* < 0.01) and 0.800 (*p* < 0.01), respectively.

**FIGURE 5:**
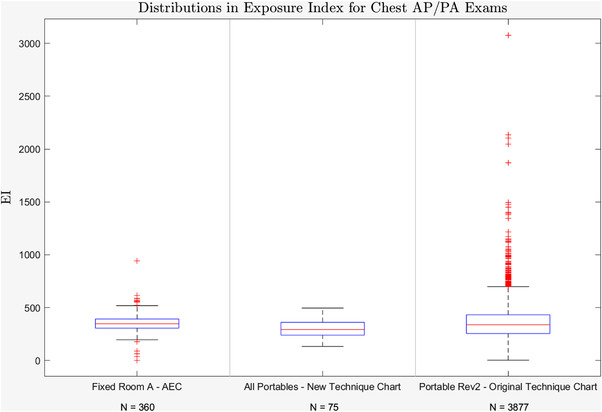
Box and whisker plot of PA chest X‐ray exposure indices for a fixed room radiographic unit (Fixed Room A ‐ AEC), the five portable units using the height/weight‐based technique chart (all portables ‐ New Technique Chart) for AP chest studies, and one portable unit that relies on a technologist to select the technique (Portable Rev2 ‐ Original Technique Chart) for AP chest studies. The boxes extend from the 25th to 75th percentile with the median indicated by a red line. The whiskers extend ±2.7σ (σ = 84.43, 80.15, and 176.73 for the Fixed Room with AEC, Portables with Updated Chart, and Portable with Original Chart, respectively) and the red crosses represent outliers

The results for variability in EI are summarized in Tables 2 and 3 for the chest and abdomen portables, respectively. Both the chest and abdomen examinations using the new technique showed less variability (*p* < 0.01) than the images acquired using a single portable unit with a programmed technique chart (“original technique chart”) which contains three size options for small, medium, and large patients. The variability was comparable to chest and abdominal examinations acquired on a single room (“Fixed Room A ‐ AEC”) chest and table Bucky (*p* > 0.05). Figure 5 illustrates the variability in EI for AP chest X‐rays acquired using the new technique chart relative to images acquired using a single fixed unit utilizing a Bucky system with AEC as well as images acquired using the old technique chart on a single portable X‐ray unit.

**TABLE 2:** Statistical summary of exposure index (EI) for portable chest anteroposterior/posteroanterior (AP/PA) images

Statistics	Portables – new technique chart	Portable Rev2 – original technique chart	Fixed room A ‐ AEC
Number of studies	75	3877	360
Mean EI	301 (282–319)^a^	365	350
Median EI	293 (262–327)^a^	339	347
Standard deviation of EI	70.1 (69.1–88.6)^a^	176.7	84.4
Skewness of EI distribution	0.206 (–0.226 to 0.607)^a^	3.04	0.686
Levene's test (*p* value)	NA	0.00001	0.2681

^a^ Determined using bootstrapping with replacement, 95%.

**TABLE 3:** Statistical summary of exposure index (EI) for portable abdomen images

Statistics	Portables – new technique chart	Portable Dart5 – original technique chart	Fixed room A ‐ AEC
Number of studies	99	200	112
Mean EI	335 (312‐358)^a^	286	398
Median EI	321 (293‐353)^a^	226	417
Standard deviation of EI	115.7 (96.2‐133.7)^a^	257.4	122.9
Skewness of EI distribution	0.65 (0.109‐1.17)^a^	4.21	–0.913
Levene's test (*p* value)	NA	0.0033	0.8626

^a^ Determined using Bootstrapping with replacement, 95%.

## 4 DISCUSSION

The findings demonstrate a relationship between the tissue equivalent thickness and patient height and weight. Although tissue equivalent thickness along a given projection is not explicitly defined by the height and weight, it can be derived from the WED with specified conversion factors. The convenience of this relationship lies in the fact that technique optimization can be carried out from the RIS/HIS data, which can be sent to the portable unit itself through the modality worklist (MWL). Although these technique charts were printed on paper, this method could be directly integrated into the portable unit to achieve automatic technique selection. This study used the EI as a surrogate for air kerma at the detector, which was then applied to calculate the necessary technique. The relationship between EI and air kerma is dependent on beam quality. The range of selected kVps, especially for portable abdominal examinations, was restricted to a smaller range than used clinically. The data collected to produce the technique chart utilized techniques similar to kVps, which helped minimizing the dependence. In addition, the use of an anti‐scatter grid, the grid characteristics, and how the EI is calculated will affect how the patient tissue equivalent thickness is calculated. When using this methodology to produce a technique chart, the authors recommend collecting data that are based on the same geometry, grid, target EI, and range of tube potentials that are currently being used clinically. This will help minimize dependencies not accounted for in this method. It has been demonstrated that systems that are of the same make and model can share a technique chart assuming similar inherent filtrations in the X‐ray tube and collimator.

EI serves as an indirect estimation of image signal‐to‐noise ratio (SNR), which is useful in benchmarking technique charts for digital detectors to minimize the contribution of noise and avoid excessive radiation exposure. Our methodology demonstrates comparable variability in EI values with respect to an AEC system. Compared to the old technique charts, which utilize three patient sizes, our method demonstrates lower variability in EI values despite both charts achieving a similar target EI on average. This methodology, combined with the development of simulated anti‐scatter grids, has the potential to improve imaging in both mobile radiography and stretcher examinations performed outside the Bucky. The distance between the focal spot and the detector must be assumed, so maintaining a consistent distance is important for reducing variability. Variability in exam source‐to‐image distance was not accessed in this study. In portable imaging, consistency in the SID may not always be achievable, though the technologist can often get to within a few centimeters of the specified distance with a tape measure. Technologists must always be aware of the distance from the X‐ray tube at which they are placing the detector.

An additional benefit of this proposed method is the ability to adjust the target EI based on patient thickness. This is desirable because maintaining SNR across variable patient sizes does not translate into consistent image quality. This is due to scattered radiation, which reduces image contrast and correlates with the thickness of the body part being imaged. If one can model changes in image contrast based on the patients’ tissue equivalent thickness and can also accurately predict a given tissue equivalent thickness based on the MWL information, then detector exposures can be tailored to different patient sizes to maintain image quality. It should be the role of the physicist and lead radiologist to determine the target EI as well as any adjustment based on patient size.

## 5 CONCLUSIONS

This study highlights a methodology to construct a technique chart for a single anatomical view from data available through the RIS/MWL, data collection of patient and examination parameters, and simple measurements of X‐ray machine characteristics. The technique chart delivers reproducible and accurate EI values and for a wide range of patient sizes (including adults and pediatrics) and could be further modified to produce variable detector exposure levels based on tissue equivalent thickness. Further investigation is necessary in optimization of variable target EI based on patient size.

## CONFLICT OF INTEREST

The authors declare no conflict of interest.

## AUTHOR CONTRIBUTIONS

Matthew Hoerner wrote most of the manuscript and did most of the data analysis. Kevin Grizzard prepared the figures and performed the remainder of the data analysis and assisted with defining the equations. Jennifer Moroz and Kevin Grizzard created the technique charts and helped organize and collect data. All authors reviewed the final manuscript and contributed to revisions.
